# Time-Use and Mental Health in Older Adults: A Scoping Review

**DOI:** 10.3390/ijerph18094459

**Published:** 2021-04-22

**Authors:** Hui Foh Foong, Sook Yee Lim, Roshanim Koris, Sharifah Azizah Haron

**Affiliations:** 1 Malaysian Research Institute on Ageing (MyAgeingTM), Universiti Putra Malaysia, Serdang 43400, Selangor, Malaysia; huifoh@upm.edu.my (H.F.F.); roshanim@upm.edu.my or; 2 Department of Dietetics, Faculty of Medicine and Health Sciences, Universiti Putra Malaysia, Serdang 43400, Selangor, Malaysia; l.sookyee@yahoo.com; 3 Faculty of Business, Economics and Social Development, Universiti Malaysia Terengganu, Kuala Nerus 21030, Terengganu, Malaysia; 4 Department of Resource Management and Consumer Studies, Faculty of Human Ecology, Universiti Putra Malaysia, Serdang 43400, Selangor, Malaysia

**Keywords:** time use, daily diary, mental health, depression, happiness

## Abstract

Time-use of older adults can be different than in earlier life, especially during the transition from pre- to post-retirement or after experiencing major life events, and the changes could affect their mental health. However, the extent and nature of such research in gerontology have not been examined to date. Therefore, this scoping review sought to map the literature on time-use and mental health in the older population to examine the extent and nature of those research activities. A scoping review was conducted using four databases—PubMed, Scopus, CINAHL, and EMBASE according to PRISMA guidelines. Data were extracted using a pretested tool to develop a descriptive analysis and thematic summary. A total of 11 articles met the eligibility criteria. Seven out of 11 studies involved cross-sectional design, while the remainder were longitudinal studies. The longitudinal studies mainly were secondary data analysis. Time-use data were mainly collected using daily diaries, and the most common mental health outcome included was depression. Only two studies did not evaluate the direct relationship between time-use and mental health. Our review has revealed studies evaluating time-use and mental health in older adults. Limitations of review and recommendations for future studies are discussed.

## 1. Introduction

The time-use study aims to examine how a person occupies time. Although identification of time-use in older adults has been labeled as an important direction, literature on how older adults spend their time still lacks, especially in Asian countries [1]. Most of the existing empirical studies are from developed Western countries. The time-use research in older adults started as early as 1997 when McLennan reported that older people aged 65 and above in Australia spent most of their time in personal care, domestic, and leisure activities [2]. Gauthier and Smeeding concluded that older adults from nine countries in Europe and North America spent more time watching television, reading, and listening to the radio after they stopped working in paid jobs [3]. In Asia, according to Punyakaew et al., older adults in a Thailand suburban community spent around 8.6 h on rest and sleep, followed by 6.3 h on leisure activities and 4.9 h on work [4].

Observation, experience sampling, and time diaries are three possible methods to collect time-use data, and each has strengths and drawbacks [5]. Observational methods are useful in collecting detailed information about time-use in a particular environment and the number of times a behavior has occurred in a small sample study; however, it requires human resources for the observation and is potentially intrusive [1]. Researchers may consider using experience sampling if they intend to minimize the typical time-use data and to know more about time-use in randomly chosen portions of time. The strength of this method is it does not require memory as this is not a recall method. However, this method does not tell patterns of activity and the frequency of activities [6]. Time diaries are the best and most common approach to collect time-use data [7]. Time diaries collected detailed information on the types of activity, the duration of activity and activity patterns with time intervals ranging from 5 min to an hour interval; however, this method has been criticized for bias to the data due to the retrospective recall [1]. Therefore, researchers suggested using larger time intervals (30 min) to improve recall accuracy [7]. Productive time-use means active lifestyle and involvement in various activities, such as physical exercise, hobbies, social, and leisure activities. Past studies have shown that practicing an active lifestyle by participating in physical activity and social activities are associated with better cognitive function [8,9], higher wellbeing [10,11], and lower depressive symptoms [12,13] in older adults.

Population aging across the world causes implications not only for the disease burden but also for the social and healthcare system [14]. For instance, the number of older people with mental disorders is expected to double by 2030, and statistics have shown that approximately 15% of older adults aged 60 and above experience mental disorders, such as depression, anxiety, isolation, or dementia [15]. Depression appears as one of the most common mental disorders in older people [16]. Previous studies have shown that good mental health in old age promotes not only quality of life [17] but also longevity [18]. There is compelling evidence that being active in old age is associated with good mental health. For example, several studies have concluded that physical activity reduces the risk of depression in older adults [19,20]. Moreover, physical activity is also associated with better optimism, life satisfaction, positive affect, and psychological wellbeing in older adults living with loneliness [21].

Activity patterns of older adults can be different than in earlier life, and the changes in time-use and activity pattern on mental health are crucial as good mental health is an important element in successful aging [22]. Moreover, activity engagement and active lifestyle remain central in several models of healthy aging, such as the successful aging model [23,24,25] and the productive engagement framework [26,27]. Since no reviews have examined this topic, a clear need exists to comprehensively review the studies of time-use and mental health in older adults. This scoping review aims to map the literature on time-use and mental health in the older population to examine the extent and nature of those research activities.

## 2. Materials and Methods

### 2.1. Research Question

The development of this scoping review followed the guidelines by Arksey and O’Malley [28], which started from developing a research question. As suggested by Richardson and colleagues, [29] the research question was determined according to the population, intervention, controls, and outcomes. This scoping review aims to resolve the following research question “What is the extent and nature of research activities on time-use and mental health among older adults?”

### 2.2. Search Strategy

Two investigators independently searched PubMed, Scopus, CINAHL, and EMBASE databases for potential studies published in journals from inception to November 2020. There was no limit on the years of publication, as long as it was published before or on 30 November 2020. The search was restricted to studies focusing on time-use and mental health in older adults. Table 1 presents the search terms used in the present review.

### 2.3. Eligibility Criteria

Table 2 shows the domain, inclusion and exclusion criteria, and rationale of the criteria. The inclusion and exclusion criteria were:

- Any study from inception until 30 November 2020;
- Studies from any country;
- Studies only in English;
- Studies only published in peer-review journals;
- Quantitative study with either experimental, longitudinal, cross-sectional, or correlational designs so that the relationship between time-use and mental health could be evaluated if the study did the analysis;
- Studies that involved human time-use and mental health involving older adults. Studies that involved both older adults and non-older adults were permitted with the condition that the involvement of non-older adults was so that the findings on older adults could be compared with non-older adults;
- Studies that collected time-use on a broad range of daily activities, but not discrete activity in isolation. Studies that collected activity engagement using a frequency scale or with “yes/no” responses were excluded as they cannot provide information on activity duration.

### 2.4. Screening

First, two investigators independently imported the potential articles from the databases into Endnote Program X5 Version. Any duplicate publications were removed manually and by Endnote Program X5. Second, the titles and abstracts were screened independently by them for suitability based on the search strategies mentioned above. Then, full-text articles were independently assessed based on the inclusion and exclusion criteria. Lastly, they performed a manual hand-search for the potential articles from the citation of the included articles. All included articles were agreed upon after discussing them with the third investigator.

### 2.5. Data Extraction

The following data were extracted by two investigators independently using a standardized, comprehensive, and piloted data extraction template—the last name of the first author and year of publication, country, study objective, study design, sample characteristics, the instrument used to measure time-use, type of mental health measured, the instrument to assess mental health, main statistical analysis, and study findings. The disagreement was resolved after consulting the third investigator. Quality appraisal of each study was not performed as this is not a compulsory procedure in scoping review [30].

### 2.6. Data Analysis

All authors read, understood and synthesized the findings. Several discussions were conducted among the study group to categorize findings into the following themes [30]; “demographic, time-use, and mental health,” “changes in time-use and mental health,” work status, types of activities, and mental health,” and “activity profiles and mental health.”

## 3. Results

### 3.1. Study Characteristics

**Figure 1:**
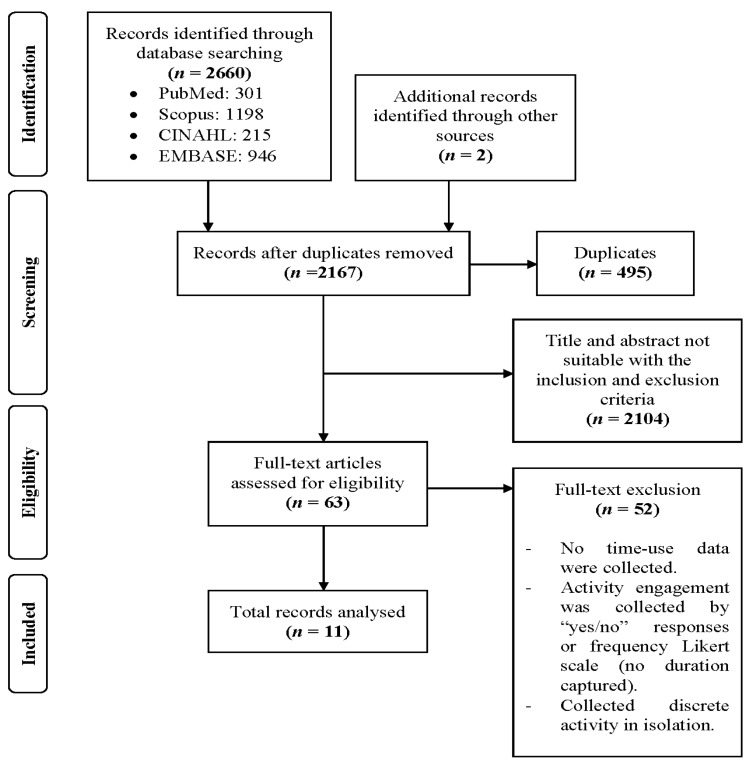
Scoping review flow chart.

Figure 1 illustrates the results from the search and study selection processes. The systematic search of electronic databases yielded 2167 potential studies with 11 articles published between 1995 and 2019 that met the inclusion criteria for this review (see Table 3). In general, the number of studies did not increase by each year except for a peak of three studies in 2018. The regions represented in the review showed that authors from the United States contributed the most studies (*n* = 6, 54.5%), followed by Australia (*n* = 3, 27.3%), Netherlands (*n* = 1, 9.1%), and one study (*n* = 1, 9.1%) had combined data from Italy, Spain, United Kingdom, France, and The Netherlands. All studies were from developed European countries and none from Asian countries.

The summary of all the included study is presented in Table 4. Six studies (54.5%) involved secondary data analysis, and the other five studies (45.5%) involved primary data analysis. Two studies (18.2%) analyzed data from the National Study of Daily Experiences in the United States. The other two studies (18.2%) drew the health and retirement study data linked with consumption and activity mail survey from the United States. One study (9.1%) analyzed data from the multinational time-use study, which combined data from five different countries; Italy, Spain, United Kingdom, France, and the Netherlands. Another study (9.1%) obtained data from a driving cessation study in Australia. In terms of study design, most of the studies (*n* = 7, 63.6%) were cross-sectional. Four studies (36.4%) had a longitudinal study design.

In terms of the study sample, 45.5% of the studies involved general community-dwelling older adults. One study (9.1%) involved older widowed women, older adults who just lost their spouse, pre-retired older workers, and older drivers. Two studies (18.2%) involved people from different age groups so that the findings on the older group could be compared with other age groups. For sample size, the studies (*n* = 3, 27.3%) with secondary data analysis had a bigger sample size (number of participants > 1000). In contrast, five studies (45.5%) utilized a sample size between 100 and 800, and three studies (27.3%) involved a sample size of less than 100. This review also found that two studies (27.3%) collected time-use and mental health data, but the authors did not examine the relationship between these two variables.

### 3.2. Time-Use and Its Measurement

The measurement of time-use is varied across all studies. Some of the instruments noted from this review were the daily diary [33], multimedia activity recall for children and adults [39], the social rhythm metric [31], activity configuration [5,34], and yesterday’s diary [35]. One similarity of all the measurements was that most of them covered time-use recall for both weekdays and weekends. In terms of data collection duration, Hahn and colleagues and Lee and colleagues took daily details for eight consecutive days of the time used to capture the type of activities and their time-use [33,38]. Some researchers chose to measure time-use on a weekly and monthly basis by asking the number of hours spent in a week and month for an activity [36,40]. Moreover, Olds and colleagues took a 2-day time-use (1 weekday and 1 weekend) [39]. Aside from collecting data about time spent on certain activities, researchers also considered other useful information, such as the location of the activity and the presence of other people [34].

Some authors collected time-use data for other purposes of analysis. For example, Prigerson and colleagues collected time-use data and generated the Social Rhythm Score used to investigate the sample’s social rhythms and lifestyle regularity [31]. Olds and colleagues examined the time-use in mature workers pre- and post-retirement [39]. They conducted time flow analytics after collecting time-use data and presented the time flow among super domains (e.g., work, transport, social, quiet time, screen, etc.) using a chord diagram [39]. Some studies also categorized selected activities based on domains. For example, Jennings and colleagues clustered a series of activities based on the physical, mental, and social domains [32]. Olds and colleagues listed activities based on transport, social, quiet time, screen, sleep, self-care, chores, physical activity, and work domains [39].

### 3.3. Mental Health and Its Measurement

Depressive symptoms were the most common mental health outcome across all the studies (*n* = 5, 45.5%) [31,36,38,39,40]. Examples of instruments used to assess depressive symptoms were the 17-item Hamilton Rating Scale for Depression [31], Center for Epidemiological Studies Depression Scale [36,40], and Depression, Anxiety, and Stress Scale [39]. Lee and colleagues measured depression using seven depression items and six items of anhedonia [38]. Three studies (27.3%) included self-rated health as the wellbeing outcome, and all of them measured this variable using a single-item self-rated health scale [36,37,40]. Three studies (27.3%) measured life satisfaction as the mental health outcome [5,34,39]. The authors used the Australian Unity Personal Wellbeing Index [39] and the Life Satisfaction Index Z [5,34] to measure life satisfaction. Three studies (27.3%) included general psychological wellbeing in their studies [33,38,39]. The psychological wellbeing measurements were varied: The short Warwick–Edinburgh mental wellbeing scale [39] and the psychological wellbeing scale [38]. Moreover, Hahn and colleagues measured the sample’s daily wellbeing by administering 14 items on different negative affects and 13 items on different positive affects with a 5-point Likert scale [33]. Tadic and colleagues measured happiness in their study using a single item for happiness with a graphical face scale [35]. Two studies (18.2%) involved cognitive function as the mental health outcome, and both measured cognitive function by administering the verbal memory test [32,40].

### 3.4. Thematic Summary

#### 3.4.1. Demographic, Time-Use and Mental Health

Adjei and colleagues examined the sex differences in the relationship between work-related time-use and stress [37]. They found that both older men and women reported higher levels of stress with increasing time-use in managing housework. Moreover, the negative relationship between paid work and stress was only found in older men [37]. Hahn and colleagues examined the differences in daily time-use and wellbeing in widowed and married older women [33]. They did not find any differences in daily time-use for most of the activities. Widowed women spent more time accompanying their children and watching television and less time sleeping than married women. No difference was noted in wellbeing between widowed and married older women [33].

Liddle and colleagues examined the role of driving status in older adults’ time-use and life satisfaction [34]. They collected driving status as one of the demographic information aside from age, gender, living situation, and the number of health conditions. They found that older adults who never drove spent more time doing charity work than retired drivers. In addition, current drivers spent more time engaging in social leisure activities than retired drivers [34]. However, retired drivers spent more leisure time in solitary than current drivers, and current drivers spent more time away from home than retired drivers. In terms of mental health, current drivers reported higher life satisfaction than retired drivers, and no difference in life satisfaction was noted between retired drivers and older people who have never driven [34].

#### 3.4.2. Changes in Time-Use and Mental Health

Changes in time-use and their association with mental health could be examined using a longitudinal design. Olds and colleagues examined the time-use, depressive symptoms, life satisfaction, wellbeing, and self-esteem of mature workers pre- (6-month before) and post- (3-, 6-, and 12-month after) retirement [39]. Retired individuals spent more time on household chores, sleeping, screen time, and quiet time during retirement. Changes in overall time-use were significantly associated with lower depression and stress, as well as higher self-esteem. Replacing working time with physical activity and sleep was associated with improvements in all measures of mental health [39]. Lee and colleagues investigated the relationships between activity diversity, wellbeing, depression, positive and negative affects by following up on a group of respondents from different age groups for 10 years [38]. The study summarized that increased activity diversity in older adults was associated with greater psychological wellbeing and positive affects and decreased negative affects compared to younger respondents [38].

#### 3.4.3. Work Status, Types of Activities and Mental Health

Tadic and colleagues examined the role of work status in the relationship between time-use and happiness in older adults [35]. Overall, non-working older adults were happier than older individuals who were working. Working older adults reported lower levels of happiness with higher time spent in administrative duties. Regardless of work status, engaging in leisure activities was associated with higher levels of happiness. In addition, working older adults reported higher levels of happiness on weekends compared to weekdays [35]. Jennings and colleagues examined the relationship between time-use in different activities (physical, mental, and social) and memory performance [32]. Results showed that physical activity was positively associated with memory performance but not social and mental activities [32].

#### 3.4.4. Activity Profiles and Mental Health

Chen and colleagues identified activity patterns and the natures of engagement of older participants by using the time-use data and correlated the activity profiles with cognitive function, depressive symptoms, and self-rated health [40]. They identified five patterns of activity—“high”, “moderate”, “low”, “passive leisure”, and “working,” as well as three natures of engagement—“full”, “partial”, and “minimal” engagement. Older adults who were in the “high” and “working” group reported better self-rated health, cognitive function, and lower depressive symptoms than those of the “passive leisure” group. Older adults in the category of “low” activity with “full” engagement reported higher levels of self-rated health than older adults in the “passive leisure” group. Older adults in the category of “moderate” activity, “high” activity, and “working” group reported lower levels of depressive symptoms only when they are fully engaged in an activity [40].

Morrow and colleagues identified the sample’s activity profiles using time-use data and explored the relationships between activity profiles, self-reported health, and depressive symptoms [36]. They categorized respondents based on five activity profiles—“low activity”, “moderate activity”, “high activity”, “working”, and “physically active”. Findings showed that older adults in “high activity”, “physically active”, and “working” groups reported better self-reported health than those in the “low activity” group. Moreover, respondents in the “low activity” group reported higher depressive symptoms than all other groups [36]. Prigerson and colleagues examined if lifestyle regularity was associated with lower levels of depressive symptoms in older adults who just lost their spouse [31] and found that lifestyle regularity was associated with lower depressive symptoms in subjects with Activity Level Index higher than 80 at 12- and 24-months post-loss [31].

## 4. Discussion

### 4.1. More Evidence on Time-Use and Mental Health in Older Adults Are Required

The study of time-use and mental health in older adults still lacks in gerontology as the number of publications remained stable and did not increase over the years. This review found only 11 studies can be included as ten papers have been excluded after the full article screening. Those excluded studies only traced activity involvement by asking the respondents to answer either “yes” or “no” or rate their frequency of participation based on a Likert-scale. No time-use data for each activity was collected. We decided to exclude them as they cannot capture the duration of activities. Therefore, the activities that older adults spent on the most and least cannot be determined. Our decision is supported by Bauman and colleagues when they argued that information of daily activities, such as the order, duration, and characteristics, is essential in the time-use study as that information is useful to determine potential health outcomes and consequences [41]. The findings from this review suggest that gerontologists can work more on the research of older adults’ time-use and its relation to mental health.

All the studies included were from Western and developed countries. None of the studies were from the Asian perspective. It reflects the Western perspective that dominates the study of time-use and mental health in older adults. Limited studies from Asian countries are a problem because perceptions and meaning of time and mental health may differ across cultures and countries [3,42]. For example, older adults spending more time providing care to grandchildren is more virtually universal in Asia than in Western Europe [43]. Moreover, Asian countries like Singapore, Japan, and South Korea exhibit many older adults who continue to work although reaching retirement age due to rapid population aging. Therefore, they still allocate more time to work compared to older adults in other countries. Consequently, the relationship between different time-use and mental health would differ across cultures and countries [44,45]. The difference in the relationship between time-use and mental health across different settings can be used to interpret how time-use pattern influences the mental health of older people. This review suggests that more time-use and mental health research in older adults is required in Asian countries. The studies are essential to gather information on the daily activities of older adults from diverse communities, cultures, and various geographical locations and how this time-use could contribute to their mental health in later life.

### 4.2. Considering Robust Research Design in Time-Use and Mental Health Study

Over half of the studies in this review applied a cross-sectional design. More longitudinal studies to examine the relationships between time-use and mental health are warranted in gerontology. Besides providing a causal relationship between independent and criterion variables, longitudinal studies are important in gerontology because it allows access changes over time and specific disease endpoints [46]. In this context, longitudinal studies provide stronger evidence on how changes in time-use could affect older people’s mental health and the types of activities that older adults could participate in for better mental health. Older adults’ daily life could change drastically during retirement due to work time that will be replaced by other activities and a series of psychological adjustments [47]. Therefore, it is crucial to examine time-use changes, particularly changes from pre- to post-retirement, and examine if these changes result in mental health changes. The relationship between changes in time-use and mental health should be examined in both Western and Asian countries due to cultural and lifestyle differences so that comparisons could be made [48,49]. By doing so, we can also understand how cultural differences in time-use could affect mental health in later life.

### 4.3. Allowing Full Richness of Time-Use Data

This review found that less than half of the studies included involved cluster analysis or latent class analytic strategy. The latent class analysis helps to simplify and integrate complex human behaviors by analyzing a number of abstract indicators that could not be directly observed [50]. The reporting of time allocation solely from time-use data is deemed to be insufficient due to the inability to make full use of the data. In time-use, latent class analysis helps capture activities as a multidimensional unit. Unique patterns that are not shown by the aggregate-level average could be discovered [51]. In other words, latent class analysis allows the full richness of time-use data to be utilized. For example, studies often assign participants to different activity levels (low, high, moderate, working, etc.) based on time-use data by cluster or latent class analysis so that the association between activity levels and mental health outcome could then be estimated. Therefore, besides reporting the average time-use for each activity, this review suggests future time-use researchers also perform cluster analysis from time-use data to identify other underlying constructs, such as activity levels (low, moderate, low, etc.) and activity patterns (regular, tedious, packed, etc.). This review also found that some studies collected time-use and mental health data but did not examine their relationships. Although there are just a few, this also reminds future studies to examine the relationship between time-use and mental health in old age to close the “critical gap” in gerontological research.

### 4.4. Incorporating Demographic Issues in Time-Use and Mental Health Studies

Demographic characteristics, particularly sex, are an important concern in gerontology. Due to different socialization in the earlier stage of life, sex differences in time-use and mental health are considered substantial issues to be considered in gerontology [52]. This is visible in Asia when men are often portrayed as the breadwinner of the family, while the women’s role is highly associated with child-raising and homemaking. Past studies have shown that there are sex differences in time-use among older adults [53]. In this review, very few studies considered the sex (and other demographic variables) differences in time-use and mental health; therefore, researchers should consider examining the sex differences in time-use and if the differences affect the mental health of older adults. Aside from sex, the roles of other demographic characteristics such as marital status and work status on the relationship between time-use and mental health should be considered too.

### 4.5. Limitations of This Study

Some limitations of this scoping review are worth noting. One of the main limitations is that this review was limited to articles that were published and written in English. This may have excluded articles that were published in other languages, and therefore underestimating the amount of evidence. Moreover, some included studies involved a small sample size and non-random sampling technique, which will affect the findings’ generalizability. Next, the present scoping review did not include studies with a qualitative design. As the time-use study also could be conducted using the observational method, and therefore underestimating those studies collected time-use data using the qualitative design. Lastly, we limited our review to studies of mental health in old age. Future reviews should also include a broad range of chronic health conditions not limited to mental health.

## 5. Conclusions

This review has provided valuable information on published studies on time-use and mental health in older people after reviewing 11 eligible studies. Although two studies did not examine the relationship between time-use and mental health, there was evidence showing that paid work, social activity, and activity diversity were linked with better mental health. Therefore, the existing evidence might support the engagement theory of aging. This review also found that older adults spent more time in passive and solitary activities during their leisure time, but successful aging is more than that as it encourages older adults to socialize with others. Findings highlight the need to continually promote an active lifestyle in old age to promote successful aging. Most importantly, this review posted several suggestions for future research on time-use and mental health: (i) although there is a substantial challenge to collect time-use data accurately across the range of daily activities, it is advisable to collect time-use data by larger time intervals (every 30–45 min) to capture the duration of each activity adequately and improve recall accuracy, (ii) other information of activity, such as with whom and where an activity was performed should also be collected to capture information on loneliness, social isolation, and social interaction, (iii) make full use of the time-use data by performing cluster analysis to generate other information, such as activity levels and patterns, (iv) measuring mental health outcome using a validated and reliable instrument, (v) consider examining the relationship between time-use and mental health using longitudinal study design and proper statistical analysis approach, and (vi) research on time-use and mental health in older adults should be given more attention in Asia and other low- or medium-income countries.

## Figures and Tables

**Table 1:** Search terms.

Variables				Target Group
activity pattern OR time OR diary OR time-use OR time budget OR yesterday diary OR time studies OR time utilization OR daily activities OR time allocation	AND	mental health OR psychological health OR psychological distress OR psychological OR psychosocial OR mental OR life satisfaction	AND	elderly OR aging OR geriatric OR over-65 OR older

**Table 2:** Inclusion and exclusion criteria.

Domain	Inclusion Criteria	Exclusion Criteria	Rationale
1. Publication year	Studies from inception until 30 November 2020	Studies published after 30 November 2020	We did a preliminary search using the search terms at Google Scholar and found that the number of papers available was not overwhelmed
2. Publication type	Studies published in peer-reviewed journals only	Studies, reports, or other materials not published in peer-reviewed journals	To ensure the academic rigor and quality of the studies
3. Research design	Quantitative studies involved experimental, longitudinal, and correlational study design	Qualitative study or any study not involved quantitative research design	To evaluate the relationship between time-use and mental health if the study did examine for it
4. Study scope/variables	Studies that involved collecting human time-use and mental health data	Studies that collected discrete activity in isolation Studies that collected activity engagement using frequency scale or with “yes/no” response	To ensure that the review question is addressed Frequency scale or “yes/no” response cannot provide information on activity duration
5. Target group	Older adults with no specific age Studies involved both older adults, and non-older adults were permitted with the condition that the involvement of non-older adults was to so that the findings on older adults could be compared with non-older adults	Studies that did not involve older adults at all	To ensure that the review question is addressed
6. Location	Any country	Not applicable	Analyzing studies from Western and non-Western countries could help researchers in gaining deeper insight into the cultural differences in older adults’ time-use and mental health

**Table 3:** Number of studies published by year.

Year	Number of Studies
1995	1
2003	1
2007	1
2011	1
2012	1
2013	1
2014	1
2018	3
2019	1

**Table 4:** Summary of studies.

Author, Y	Country	Study Objective	Study Design	Sample Characteristics	Instrument Used to Measure Time Use	Type of Mental Health Measured	Instrument to Assess Mental Health	Main Statistical Analysis	Study Findings
1. (Prigerson et al. 1995) [31]	United States	To determine if high regularity in the timing of daily activities was protective against depressive symptoms among older adults soon after spousal death	Cohort study The regularity of daily activities was assessed at 3 months post-loss; depressive symptoms were measured at 3, 12, and 24 months post-loss	47 older adults aged between 60 and above who just lost their spouse; The distribution of the sample by sex and description of sample’s age was not provided	Time use measured by using the social rhythm metric (SRM); The instrument is diary-like in form, requiring the subject to provide daily details of the time of day at which each event occurred; Data for each week were then analyzed to come out with an SRM score; The score lies on a continuum between 0 and 7, with 0 representing greatest irregularity and 7 greatest regularity	Depressive symptoms	17-Item Hamilton rating scale for depression	Multiple regression	Baseline SRM was not associated with severity of depression at 12- or 24-month post-loss; For those with activity level index (ALI) scores of 80 or above, lifestyle regularity was negatively associated with depressive symptoms at 12-month post-loss; In subjects with ALI scores of 90 or above, baseline lifestyle regularity was associated with lower levels of depressive symptoms at 24-month post-loss
2. (Jennings and Darwin, 2003) [32]	United States	To evaluate the relationship between daily activities and memory performance in older adults	Cross-sectional study	Group 1:29 older adults, aged from 69 to 93 y (mean age = 78.1); Group 2:30 undergraduate students, aged 18 to 20 y (mean = 18.70); Distribution of respondents by sex was not provided	Participants were asked to indicate how many hours per week and how many weeks per month they participated in a variety of physical activities, mental activities, and social activities	Memory performance	California verbal learning test	Independent sample *t*-test	Time-use in physical activity was positively associated with memory performance; No association was noted between time-use in social and mental activity with memory performance
3. (Mckenna, Broome, and Liddle, 2007) [5]	Australia	To describe the time-use profile and role participation in community-dwelling older adults; to analyze if time-use and role participation changed with increasing age, and to examine the relationship between role participation and life satisfaction	Cross-sectional study Secondary data analysis of a driving cessation study	Total sample = 195 community-dwelling older adults (81 men and 114 women); Majority of them were aged from 65–74 y old (*n* = 94), followed by 75–84 y (*n* = 79), and 85+ y (*n* = 22)	Activity configuration was used to record time-use retrospectively; Participants were asked to recall their activities from the past week in half-hour intervals	Life satisfaction	Life satisfaction index-Z	ANOVA, chi-squared analysis, independent sample *t*-test, linear regression	Participants spent most of the time on sleep, followed by solitary activities and social leisure; The most common roles were friend, family member, and home maintainer; Participants aged 75 y and older spent significantly more time on solitary leisure and less time on paid work and transport compared to those aged 65–74 y; Role maintenance was significantly related to greater life satisfaction in participants aged 75–84 y; This study did not examine the relationship between time-use and life satisfaction
4. (Hahn, Cichy, Almeida, and Haley, 2011) [33]	United States	To compare daily time-use and daily wellbeing in widowed and married women	Cross-sectional study; Secondary data analysis; Data source: second wave (2004–2006) of National study of daily experiences	75 widowed women (mean age = 72.4 ± 6.67) and 125 married women (mean age = 70.0 ± 5.97)	Respondents were asked about the daily time spent for the following activities: interacting with children, performing household chores, doing work or school work, relaxing or doing leisure activities, watching TV, volunteering, giving or receiving unpaid assistance, giving or receiving emotional support, providing help to someone with a disability, and sleeping; Daily time use was collected for 8 consecutive days	Daily wellbeing	Respondents were asked how often during the past day they experienced 14 different negative emotions and 13 different positive emotions with a Likert scale ranging from 0 (none of the time) to 4 (all of the time)	ANCOVA, independent samples *t*-test	No difference in daily activity time-use for most of the activities between married and widowed women; Widowed women spent more time accompanying their children and watching television and spent less time sleeping than married women; No difference was noted in wellbeing between widowed and married older women
5. (Liddle, Gustafsson, Bartlett, and Mckenna, 2012) [34]	Australia	To examine the impact of driving status on time use, role participation and life satisfaction in older adults	Cross-sectional study	137 current drivers (mean age = 73.2 ± 6.1), 56 retired drivers (mean age = 78.7 ± 6.7), and 41 people who have never driven (mean age = 76.2 ± 6.2); The distribution of the study sample by sex was not reported	Time use was measured using activity configuration; Participants were asked to recall activities from the past week in half-hour intervals, including the location of the activity, presence of other people and subjective classification of the activity	Life satisfaction	Life satisfaction index Z	ANOVA, chi-squared, multiple regression, logistic regression	Those who have never driven spent more time doing charity activities than retired drivers; Current drivers spent more time in social leisure activities than retired drivers; However, retired drivers spent more time in solitary leisure than current drivers; Current drivers spent more time away from home than retired drivers; Current drivers had higher life satisfaction than retired drivers; No difference in life satisfaction between retired drivers and people who have never driven; This study did not examine the relationship between time-use and life satisfaction
6. (Tadic, Oerlemans, Bakker, and Veenhoven, 2013) [35]	The Netherlands	To examine the role of current work status in the relationship between time-use and happiness	Longitudinal study; Data on time use and happiness were collected monthly over three y	381 men and 198 women with mean age = 65.3 ± 7.78	Respondents were asked to fill in “yesterday ‘s diary”, once every month throughout 2006–2008 to capture time use in activities over weekdays and weekends; “yesterday’s diary” was built based on the day reconstruction method	Happiness	A single item for happiness, with a graphical faces scale ranging from 1 (extremely unhappy) up to 10 (extremely happy)	Hierarchical linear modeling, multilevel modeling	Non-working older adults were happier than working older individuals; Paid and voluntary works were associated with higher happiness; Administrative duties were associated with lower happiness in older adults with a paid job; Regardless of work status, relaxing activities were associated with higher happiness; Older adults with paid jobs reported higher levels of happiness on weekends compared to weekdays
7. (Morrow-Howell et al. 2014) [36]	United States	To identify activity profiles and explore the relationships between activity profiles and wellbeing outcomes	Cross-sectional study Secondary data analysis; Data source: 2008 and 2010, as well as the 2009 Health and retirement study consumption and activities mail survey	1942 men, 2743 women with mean age = 69.5 ± 8.91	36 activity measures—measured continuously—hours per week, hours per month	Self-reported health; Depressive symptoms	Single-item self-reported health scale with Likert scale from 1 (excellent) to 5 (poor); Center for Epidemiologic Studies—Depression (CES-D)	Generalized linear modeling, multinomial logistic regression, multiple linear regression, general mixture model	Five activity profiles were identified: “low activity,” “moderate activity,” “high activity,” “working,” and “physically active; ” Older adults in “high activity” “physically active” and “working” groups had better self-reported health than those in the “low activity” group; Older adults in the “low activity” group reported higher depressive symptoms than all other groups
8. (Adjei, Jonsson, and Brand, 2018) [37]	Italy, Spain, UK, France and the Netherlands	To examine the associations between work-related time use, social time use and self-rated health; To examine if stress mediated the relationship between work-related time use activities on self-reported health	Cross-sectional study; Secondary data analysis Data source: Multinational time use study (WTUS, version W53)	11,168 men (mean age = 72.4 ± 5.01) and 14,295 women (mean age = 73.1 ± 5.13)	Time use was collected by self-administered diary; Respondents reported the total time spent on 41 activities over a 24 h period in 5-, 10- or 15-min intervals; Respondents in France, Italy and Spain reported the time use during a randomly assigned day in a week; Respondents from the UK filled in the diary for two days (weekday and weekend); In the Netherlands, respondents reported their time use activities for seven consecutive day	Self-rated health; Stress	One-item self-reported health scale with Likert scale ranging from 0 (poor) to 3 (very good); Time pressure was measured by asking respondents, “Would you say you always feel rushed even to do the things you must do, only sometimes feel rushed, or almost never feel rushed?” with Likert scale ranging from 1 (never) to 3 (always)	Pearson’s correlation, linear structural model	Housework was associated with higher stress in both older men and women; Paid work was associated with lower stress only in older men; Social activities were associated with better self-rated health, but no association was found with stress; Stress did not mediate the association between housework, paid work and self-reported health
9. (Lee et al. 2018) [38]	United States	To examine the associations between activity diversity and psychological wellbeing in people from different age group; older adults (60–74 y), middle-aged adults (35–59 y), and younger individuals (24–34 y)	10 y longitudinal study; Secondary data analysis; Data source: National survey of daily experiences (1996–1997 and 2006–2007)	793 (349 men, 444 women) individuals aged from 24 to 74 y at baseline (mean age = 46.71 ± 12.5)	8-day daily diary was administered to participants to capture their daily experiences, including daily time use; Seven activities were captured: paid work, with children, doing chores, on leisure, in physical activities, on formal volunteering, and giving informal help to people who do not live with respondents	Psychological wellbeing; Depression positive and negative affect	The psychological wellbeing scale 7 items of depressive affect and 6 items of anhedonia; Positive and negative affect scales	Multilevel models, residualized gain models, ANCOVA-type regression model	Older adults who engaged in more diverse activities reported higher psychological wellbeing than older adults who engaged in less diverse activities; Longitudinally, increased activity diversity over 10 y was associated with the increases in positive affect; Compared with younger individuals who increased activity diversity, older adults who increased activity diversity reported smaller decreases in psychological wellbeing, greater increases in positive affect, and greater decreases in negative affect
10. (Olds et al. 2018) [39]	Australia	To determine the effects of activity changes after retirement on mental health	Longitudinal study; Data on time use, physical health, mental health and sociodemographic characteristics were gathered 6 months before and at 3-, 6- and 12-month post-retirement	54 women and 51 men with pre-retirement mean age = 62.3 ± 4.3 y and post-retirement mean age = 63.4 ± 4.3 y	Time use in activities was collected by using multimedia activity recall for children and adults (MARCA); The MARCA was administered at each time point on two occasions, each time recalling the two previous days, one weekday and one day on the weekend	Depression, anxiety, and stress Wellbeing Life satisfaction self-esteem	Depression, anxiety, and stress scales; The short Warwick–Edinburgh mental wellbeing scale Australian unity personal wellbeing index The Rosenberg self-esteem scale	Regression analysis, compositional isotemporal substitution models	Work time flowed mainly to household chores, sleep, screen time and quiet time after retirement; Changes in overall time use were significantly associated with lower depressive symptoms and stress, as well as higher self-esteem; Replacing work time with physical activity and sleep was associated with improvements in all measures of mental health
11. (Chen, Putnam, Lee, and Morrow-Howell, 2019) [40]	United States	To examine the relationships between activity, health, and nature of engagement in older adults	Cross-sectional study Secondary data analysis Data source: 2010 and 2012 Health and retirement study linked with 2011 consumption and activity mail survey	3516 women and 2528 men with mean age 64.4 ± 10.37 y	33 items that captured a wide range of activities that involved varying degrees of physical, cognitive, and social engagement; Time use for each activity was divided into three levels; low, medium, and high	Cognitive function Depressive symptoms Self-rated health	Numbers of words recalled immediately and delayed The modified version of the Center for Epidemiologic Studies-Depression scale Single item of self-rated health with Likert scale 1 = excellent to 5 = poor	Latent class analysis, linear regression	Five patterns of activity (high, medium, low, passive leisure, working) and three nature of activity engagement (full, partial, minimal) were identified; High and working groups, compared to the passive leisure group, showed better health and cognition outcomes; Older adults in the category of “low” activity with “full” engagement reported higher levels of self-rated health than older adults in the “passive leisure” group; Older adults in the category of “moderate” activity, “high” activity, and “working” group reported lower levels of depressive symptoms only when they fully engage in the activity

## Data Availability

The data that support the findings of this study are available from the corresponding author, upon reasonable request.
